# Nano-fabrication of molecular electronic junctions by targeted modification of metal-molecule bonds

**DOI:** 10.1038/srep14431

**Published:** 2015-09-23

**Authors:** S. Hassan M. Jafri, Henrik Löfås, Tobias Blom, Andreas Wallner, Anton Grigoriev, Rajeev Ahuja, Henrik Ottosson, Klaus Leifer

**Affiliations:** 1 Applied Materials Science, Department of Engineering Sciences, Uppsala University, Box 534, Uppsala SE-75121, Sweden; 2 Department of Electrical Engineering, Mirpur University of Science and Technology, Mirpur Azad Jammu and Kashmir 10250, Pakistan; 3 Department of Physics and Astronomy, Uppsala University, Box 516, Uppsala SE-75120, Sweden; 4 Department of Chemistry - BMC, Uppsala University, Box 576, Uppsala SE-75123, Sweden; 5 Applied Material Physics, Department of Materials and Engineering, Royal Institute of Technology (KTH), Stockholm SE-10044, Sweden

## Abstract

Reproducibility, stability and the coupling between electrical and molecular properties are central challenges in the field of molecular electronics. The field not only needs devices that fulfill these criteria but they also need to be up-scalable to application size. In this work, few-molecule based electronics devices with reproducible electrical characteristics are demonstrated. Our previously reported 5 nm gold nanoparticles (AuNP) coated with ω-triphenylmethyl (trityl) protected 1,8-octanedithiol molecules are trapped in between sub-20 nm gap spacing gold nanoelectrodes forming AuNP-molecule network. When the trityl groups are removed, reproducible devices and stable Au-thiol junctions are established on both ends of the alkane segment. The resistance of more than 50 devices is reduced by orders of magnitude as well as a reduction of the spread in the resistance histogram is observed. By density functional theory calculations the orders of magnitude decrease in resistance can be explained and supported by TEM observations thus indicating that the resistance changes and strongly improved resistance spread are related to the establishment of reproducible and stable metal-molecule bonds. The same experimental sequence is carried out using 1,6-hexanedithiol functionalized AuNPs. The average resistances as a function of molecular length, demonstrated herein, are comparable to the one found in single molecule devices.

Tailor-designed molecules are promising candidates as building blocks for next generation electronics due to their size, packing density and the fact that they can be tailored with unlimited variation in their electronic properties^1^,^2^. Electronic structure and conductance of a single molecule can be determined experimentally by techniques^3^,^4^, such as scanning tunneling microscope break-junction (STM-BJ)^5^,^6^, conductive atomic force microscopy^7^ and mechanically controlled break-junctions (MCBJ)^8^,^9^. The electrical properties of molecular assemblies can also be measured through formation of self-assembled monolayers on metallic substrate which provides the back-contact and where the top contact has been made either by using liquid metal electrode^10^ or by depositing metal on top of insulating nanopores with molecules^11^ or by cross bar structure^12^.

A strong emphasis also remains on the utilization of gold electrodes with nanogaps^13^ in the process of molecular device fabrication as it provides an opportunity not only to electrically characterize at the single-molecule level^14^ but also allows for the construction of molecular devices such as transistors^15^, sensors^16^,^17^ and diodes^18^. With this fabrication technique, the electrical signature is related to the response of few molecules whereas in specialized laboratory setups such as MCBJ and STM-BJ, single molecule responses are achieved. However this setup provides the opportunity to fabricate reproducible few molecular electronic devices using standard clean room process. The devices are portable. Such nanogaps can be prepared by electron beam lithography, focused ion beam cutting, electromigration and evaporation^19^. However, a stable gap formation at room temperature between gold electrodes with a size comparable to the length of a molecule has not been shown due to self-diffusion of gold atoms at the surfaces^20^. Stable nanogaps are usually in range of few 10 nm and above^21^,^22^, thus, the utilization of such gaps for contacting molecules requires bridging the nanogap by metallic nanoparticles (NPs) which are trapped by application of either high frequency AC electrical field^23^ or external magnetic field^24^.

Molecules can attach themselves to metallic surfaces either by physisorption^25^ or by chemisorption through anchoring groups such as thiol (−SH)^3^,^14^,^23^, amino (−NH_2_)^26^, selenol (−SeH)^27^ or a series of other functional groups. Thus, it is a major challenge to bring molecules in a reproducible way between stable, *i.e*., 10 nm or wider nanoelectrode gaps. The binding of the anchoring groups to the metal surfaces plays a major role^28^ and molecules terminated on two places with anchoring groups can be introduced into the electrode gap either by the coating of electrode surfaces^23^,^29^ or functionalizing nanoparticles^14^.

In the devices, covalent coupling at both ends of the molecules to the electrode surface and nanoparticles is believed to be essential for stable and reproducible electrical characteristics and this requires anchoring groups on both sides of the molecules^13^,^28^. However, it is non-trivial to coat uneven electrode surfaces or gold nanoparticles with molecules having free-standing thiol anchor groups due to backbiting and formation of disulfide bonding^29^. Up till now, chemisorbed junctions at both end of the molecules were achieved either by formation of dimers and trimers^14^ or by place exchange^30^. Reproducibility of a device is a major problem for molecular devices fabricated from trapping of dimers and trimers.

A third contact scheme was developed that relies on the fabrication of AuNPs (5 nm in diameter) that are functionalized by alkanedithiol molecules, where the outer thiol groups are protected by triphenylmethyl (trityl) protective groups as confirmed through series of one- and two- dimensional NMR spectroscopy techniques^31^. Once these AuNPs are trapped in the nanoelectrode gap (Fig. 1a), a deprotection solution removes the protective group so that the now unprotected outer thiol group can attach to an adjacent gold surface (Fig. 1b). During proof of concept studies on one device, a strong increase in conductivity of the junction could be observed once the covalent bond is established^31^. Molecular vibrational signatures in the same devices have been observed using inelastic electron tunneling spectroscopy (IETS). Vibrational peaks are identified and number of molecules taking part in electron conduction through nanoparticle nanoelectrode bridge platform are estimated^32^. So far the reason for the strong increase in conductivity could not be elucidated as well as it has not been clear if this contact scheme could lead to an improvement of the device reproducibility. Here, in a systematic assessment of the electrical properties of such junctions, we obtain a significant improvement of reproducibility of electrical measurements from this nanoelectrode-molecule-nanoparticle bridge platform. This is the first time that a large number of reproducible measurements are carried out on nanoparticle-nanoelectrode bridge platform. By density functional theory (DFT) calculations the variations of resistance can be explained as a change in chemical bonding and molecule. The AuNP spacing obtained in the DFT calculations are verified by transmission electron microscopy.

## Results and Discussions

**Figure 1:**
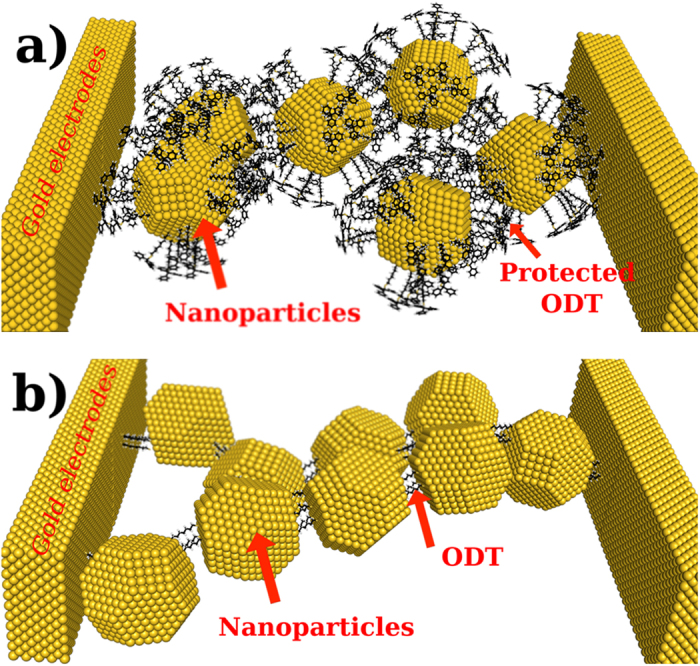
Schematic drawing of (a) dielectrophoretically trapped ω-trityl protected 1,8-octanedithiol-coated gold nanoparticles, and (b) the junction after removal of the trityl protective groups by usage of an acidic deprotection solution resulting in formation of chemisorbed junctions at both ends of 1,8-octanedithiol.

**Figure 2:**
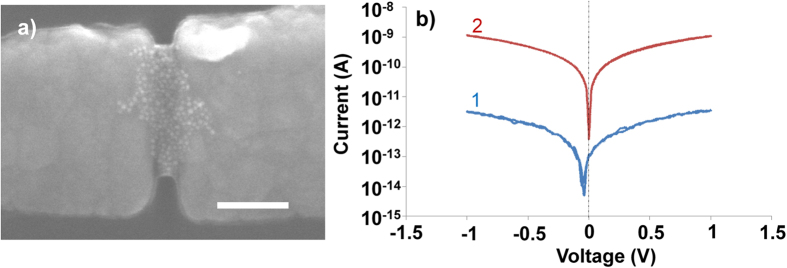
(**a**) Scanning electron image of ω-trityl protected 1,8-octanedithiol coated nanoparticles trapped in between nanoelectrode setup, and (**b**) current-voltage (I–V) response of the same device after (1) trapping and (2) after removal of trityl protective groups from the ω-end of 1,8-octanedithiol. The zero offset in curve 1 is due to the instrument and measurement system.

In Fig. 2a, we show a scanning electron microscope image of a nanoelectrode device containing trapped ω-trityl protected 1,8-octanedithiol coated gold nanoparticles (5 nm in diameter) in between two nanoelectrodes (Schematic is shown in Fig. 1a). The gap size of the nanoelectrodes is 19 nm, thus at least three functionalized nanoparticles are required to bridge the gap. The nanoelectrodes are 70 nm high and 100–150 nm wide, thus, we always trap a number of nanoparticles which might act as a 2D or realistically 3D networks of nanoparticles, where array effects should be considered in the interpretation of the results^33^. After trapping we have measured conduction response of our molecular electronic devices by applying positive and negative bias.

### 1,8-octanedithiol in nanoparticle-nanoelectrode bridge platform

In Fig. 2b, the current-voltage (I-V) characteristics of a typical device are plotted. The device consisting of ω-trityl protected 1,8-octanedithiol, demonstrates high resistance at low bias (<0.1 V) around 325 GΩ. The total calculated length of ω-trityl protected 1,8-octanedithiol that was attached on the surface of nanoparticles is 2.45 nm, the conduction mechanism should be coherent nonresonant tunneling where the transmissivity has exponential dependence on the length of the molecules and is independent of temperature^3^. Current fluctuations (Fig. 2b), can either occur due to mobility of molecules attached via thiol bonds^34^ or due to formation of kinks in the molecular chains, *i.e*., *gauche* conformers that simplistically could be seen as defects on an otherwise all-*anti*-alkane chain^35^. Higher bias voltages also induce current fluctuations as it can influence the metal-molecule interface and give conformational changes in the molecules^23^,^36^.

The trityl protective groups are removed by immersing the platform in an acidic deprotection solution (5 mL trifluoroacetic acid, 5 mL dichloromethane and 0.5 mL triethylsilane) for 20 min to establish chemisorbed junctions at both ends of the 1,8-octanedithiols thereby creating an AuNP-molecule-AuNP network bonded to the gold nanoelectrodes as shown in Fig. 1b. The electrical characterization was carried out after removing the platform from the solution, rigorously washed with de-ionized water and blow-dried with N_2_. We have plotted current-voltage characteristics after removal of protecting groups in Fig. 2b. The devices demonstrate non-linear I–V response and almost 300 times higher conduction after formation of chemisorbed junctions. We have measured resistance of this device around 1.2 GΩ at 0.1 V, which is reduced to 977 MΩ at 0.7 V. In the literature, different measurement techniques demonstrate a large variation in the measured and calculated resistance of single 1,8-octanedithiol molecule^37^. The measured resistance of 1,8-octanedithiol in self-assembled junctions is around 900 ± 50 MΩ^38^, which can be reduced to 128 ± 5 MΩ when these measurement are corrected for Coulomb blockade effect^37^. In break junction experiments, resistance value of 1,8-octanedithiol has been measured around 51 ± 5 MΩ^5^. Theoretically the simple estimate predicts resistance of 1,8-octanedithiol are 38.5 MΩ^39^ as compared to 150 MΩ from DFT calculations^38^. It should be noted that the conduction mechanism in this setup is due to coherent nonresonant tunneling of electrons^40^ and there is no change in conduction mechanism though octanedithiol with the protection group and after removal of the protection group at these applied bias voltages due to the large HOMO-LUMO gap of 1,8-Octandithiol^41^.

**Figure 3:**
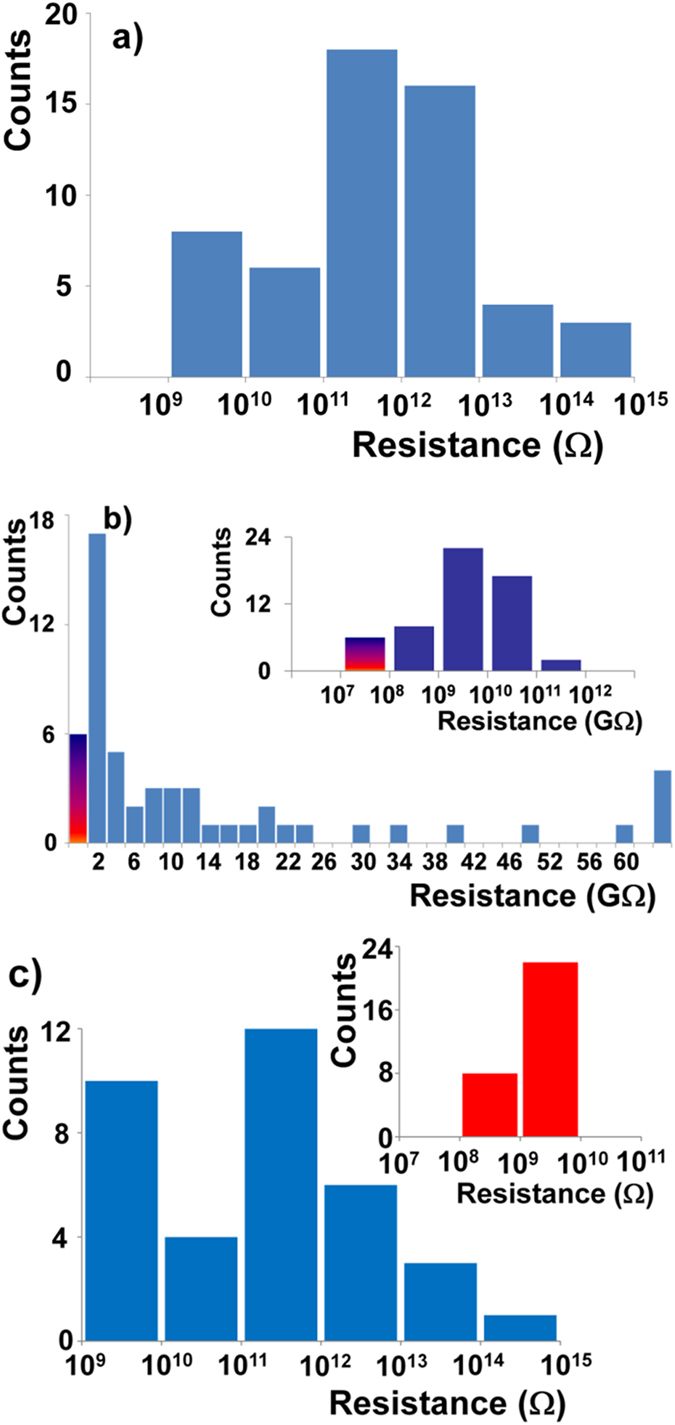
(**a**) Log-normal resistance histogram of devices having ω-trityl protected 1,8-octanedithiol in nanoparticle-nanoelectrode bridge platform (**b**) Linear scale resistance histogram (bin size = 2 GΩ) of 1,8-octanedithiol in nanoparticle-nanoelectrode bridge platform after removal of trityl protecting group and establishing a chemisorbed metal-molecule junction (inset log-normal distribution of the measured resistances). The shaded line indicates the number of devices that have demonstrated resistances lower than 100 MΩ after removal of protecting trityl groups. Inset: log-normal resistance histogram of the devices after deprotection. (**c**) Log-normal resistance histogram of devices before removal of trityl group from ω end of 1,8-octanedithiol in nanoparticle-nanoelectrode bridge platform , that shown resistance variation between 0.1 GΩ and 10 GΩ after removal of protection groups and formation of chemisorbed junctions at both ends of ODT (inset).

To demonstrate the reproducibility in our devices based on 1,8-octanedithiol molecules we have plotted resistance histogram at low bias (100 mV) of these devices before and after removal of the trityl protective groups from the outer end of 1,8-octanedithiol (Fig. 3). Considering all 55 devices as plotted in Fig 3a., the low bias resistance varies over six orders of magnitude in devices having ω-trityl protected 1,8-octanedithiol. From Fig. 3a we obtain that most of devices have demonstrated resistances in between 10 GΩ to 10 TΩ and the geometric mean resistance (from log normal fit) is found to be 470 GΩ. The large variation in resistance histogram might be attributed to the physisorbed metal-molecule junctions^28^, the number and arrangement of trapped nanoparticle and variation in contact geometries^42^. DFT calculations have shown that before deprotection, in such partially physisorbed junctions, a large spread of the resistance histogram is possible^23^. The resistance histogram after removal of the trityl groups (Fig. 3b) demonstrates most of devices (80%) have resistances in the range 100 MΩ to 30 GΩ. The devices demonstrate resistance 10 GΩ and above, SEM observations reveal that we have managed to trap very few nanoparticles in between the nanoelectrodes. In supplementary Figure S1, we have shown SEM image of a device with corresponding current-voltage (I–V) response. For six devices, we have measured resistance between 1 MΩ to 100 MΩ as shown in shaded bar in Fig. 3b, where we believe partial fusion of nanoparticles likely has occurred, resulting in lower device resistance (supplementary Figure S2). However, taking into account the device resistances of all 55 devices, the geometric mean resistance of these devices is 3.06 GΩ from log_10_ normal distribution of the histogram (peak value = 9.486 with standard deviation of 0.98 and standard error of mean is 0.13), which is an equivalent resistance of three to four 1,8-octanedithiol molecules connected in series. As the effective size of molecule-coated gold nanoparticles is between 6 nm to 6.4 nm, three nanoparticles in series are required to bridge a 19 nm nanogap between nanoelectrodes. This results in at least four 1,8-octanedithiol molecules in a shortest single current path.

Considering a resistance of 1,8-octanedithiol of about 0.9 GΩ^38^ and multiple pathways for electron conduction both in parallel and in series, it can be estimated that the resistance of such devices easily can vary between 0.1 GΩ to 10 GΩ due to different configurations of the molecular junction and current pathways. The difference of the resistance histograms between the protected 1,8-octanedithiol molecules and the unprotected 1,8-octanedithiol molecules in the nanoparticle-nanoelectrode platform becomes even clearer, if one considers only the devices that are within a resistance interval. As already observed in Fig. 3b, the histogram clearly peaks on a linear resistance scale. By selecting only the unprotected 1,8-octanedithiol devices fulfilling the above criterion, we obtain a yield of 55%. Of course, the histogram using these devices clearly peaks and is very narrow (inset Fig. 3c). In contrast, when plot the resistance histogram of the very same devices, but before deprotection, the resistances still spread over 5–6 orders of magnitude as shown in Fig. 3c. This demonstrates that using the method of initially protecting 1,8-octanedithiol functionalized Au-NPs and then deprotection of the ODT results in reproducible devices with a yield of 55%.

### Theoretical Simulation of Alkanedithiol Molecules in Nanoparticle-Nanoelectrode Bridge Platform

**Figure 4:**
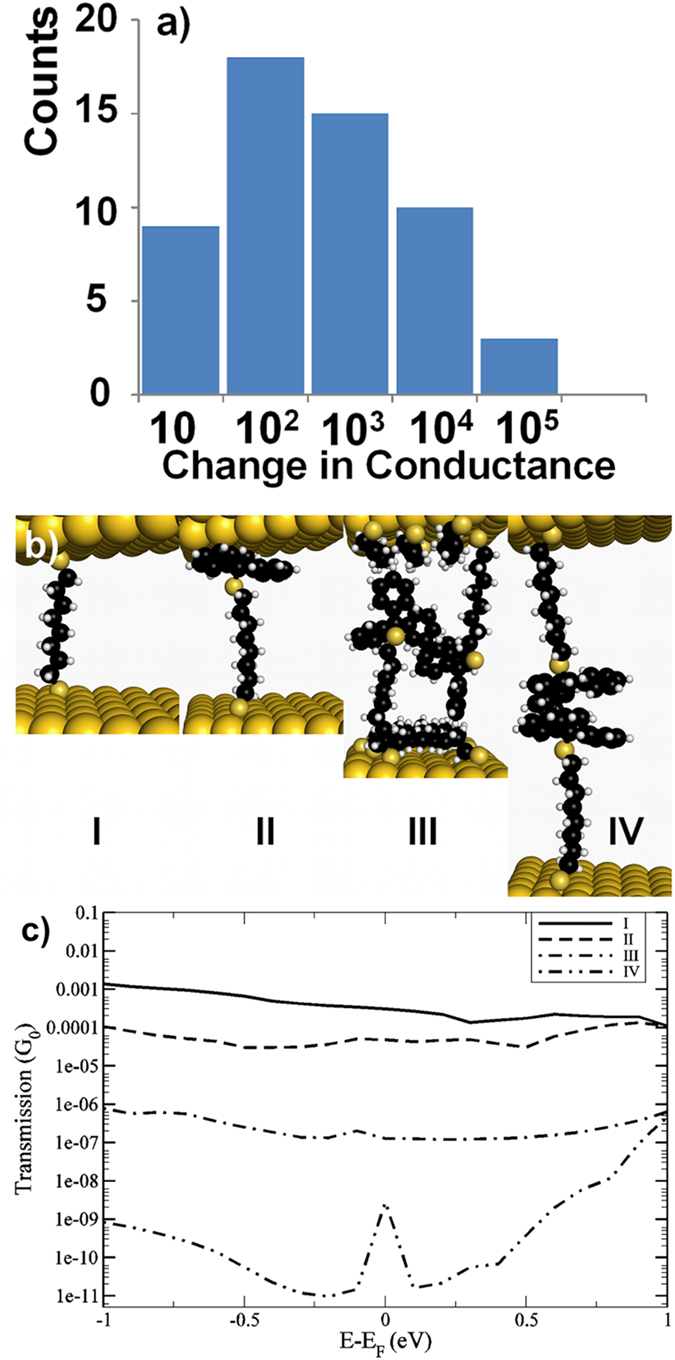
(**a**) Change in conductivity of devices upon removal of trityl protective group, after removal of trityl protective group chemisorbed junctions established at both ends of the 1,8-octanedithiol, and (**b**) atomic configuration considered in theoretical simulation I) 1,8-octanedithiol chemisorbed at two nearby gold surfaces II) ω-trityl protected 1,8-octanedithiol chemisorbed at one end and physisorbed at other end. III) ω-trityl protected 1,8-octanedithiol attached to the both gold surfaces in presence of surface layer of backbiting 1,8-octanedithiol on both gold surfaces IV) ω-trityl protected 1,8-octanedithiol attached to both electrode surface at maximum separation, and (**c**) zero-bias transmission for the four considered setups.

Another important aspect of this study is the change in resistance before and after removal of the trityl protective groups. We have summarized the ratios of the conductance before and after removal of trityl protective groups in Fig. 4a, changes in the conductance of 2–3 orders of magnitude are observed for most of the devices. To understand the change in the conduction before and after deprotection we have modeled various probable molecular configurations as shown in Fig. 4b.

The theoretical results demonstrate that the increased distance between the two adjacent gold surfaces, due to the presence of the protective groups, is mainly responsible for the decrease in conductance. A number of molecules are bent to the NP surface due to backbiting and, due to their size, the protective groups cannot penetrate the layer of these backbiting molecules^31^. Therefore this layer will act as an extra effective thickness. The ratio of back-biting versus free-standing ω-trityl protected octanedithiol molecules could be estimated as 1:4 through usage of a series of one- and two-dimensional NMR spectroscopic techniques. When the protective groups are removed the system will rearrange and our results have indicated that the terminal sulfur atoms can bind directly to an adjacent gold surface. Therefore, the distance between adjacent gold nanoparticles will be the length of the 1,8-octanedithiol molecule, and as a consequence, there is a 6–10 Å reduction in distance between the surfaces of two adjacent AuNPs. The results from the DFT calculations are summarized in Fig. 4c and Table 1. For a single 1,8-octanedithiol chain linked to the gold surface via thiols in both ends (setup I in Fig. 4b), we have obtained a conductance of 30.5 × 10^−5^ G_o_, (G_o _= 2e^2^/h) comparable to other theoretical studies^39^. In the case of ω-trityl protected 1,8-octanedithiol molecules (setup II in Fig. 4b), we have obtained a single molecule conductance 15 times lower than that of the unprotected molecule. The decrease in conductance found in the theoretical calculations can be accounted for by the increase in distance between gold surfaces from 15 Å to 18  Å to accommodate the trityl groups. Assuming that the tunneling decay factor of the alkane chain is 0.7 Å^−1^ 43,44,45, this would result in a decrease in conduction of about 10 times with this increase in distance.

**Figure 5:**
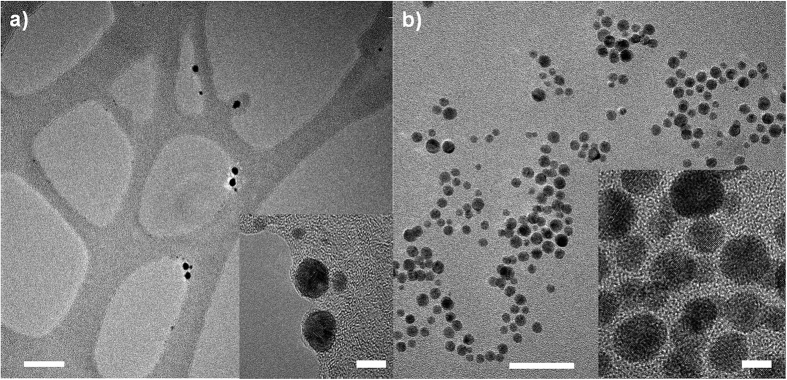
Transmission electron microscope images of (a) ω-trityl protected 1,8-octanedithiol coated nanoparticles, scale bar: 20 nm (Inset: high resolution image with a scale bar of 5 nm) and (b) after removal of the trityl protective groups from ω-end of 1,8-octanedithiol-coated nanoparticles in solution, scale bar: 20 nm (Inset: high resolution image of gold nanoparticles with a scale bar of 5 nm).

The trapped AuNPs are in deprotection solution at room temperature and are highly moveable. Thus, the AuNPs can be expected to rearrange during deprotection and accommodate to a slightly smaller average inter-AuNP distance. Such observation can be seen in transmission electron microscope images before and after removal of trityl protective groups from 1,8-octanedithiol coated gold nanoparticles as shown in Fig. 5. Still, the observed change in conductance was much greater than found by setup II, thus, this configuration in the junction cannot explain the experimental findings. Instead in a second attempt, it is assumed that the gold nanoparticles cluster together so that protective groups bind/interact with each other head to head (setup IV in Fig. 4b). This structure has a conductance of roughly 10^−9^ G_o_, hence about 6 orders of magnitude smaller than the deprotected molecule, such increase in the conductivity is either not observed experimentally after deprotection as shown in Fig. 4a. Instead in setup II and setup IV, we have identified two extreme configurations that are possible in the junction, and the measured spread of conductance indicate that experimental configuration is somewhere in between these two.

To go further in the investigation it is necessary to take into account the backbiting molecules since they might affect the distance between the electrodes when the protected molecules are clustering together. The results from this calculation (setup III in Fig. 4b) shows a zero-bias conductance of 1.3 × 10^−7^ G_o_ , i.e. a decrease with three orders of magnitude compared to the octanedithiol chain. This is in good agreement with most observed values found in the histogram in Fig. 4a. Our results suggest that it is not the protective group itself that is responsible for the experimental measured decrease in conductance, but the increased distance between gold surfaces due to the size of the protective groups and the extra effective thickness added by the layer of molecules on the surface.

### Inter-nanoparticles distances before and after removal of protection groups from Alkanedithiols

These calculations are supported by TEM observation as shown in Fig. 5, where we have shown the change in distance between gold nanoparticles before and after deprotection. It is difficult to estimate different inter-nanoparticle distances due to projection effects between adjacent nanoparticles, which appear especially, when small and big NPs interface (inset of Fig. 5b). Supposing that the NPs are lying on top of a flat carbon film of the TEM grid, we can though suppose in neighbored NPs of similar size that projection effects are largely absent. Thus, the measured interparticle spacing is likely to present the real interparticle spacing for NPs of close to the same size. The ω-trityl protected 1,8-octanedithiol coated AuNPs are shown in Fig. 5a, where the inter-nanoparticles distances vary between 1.4 nm and 5 nm with an average distance between two gold nanoparticles in the order of 2.8 nm (inset Fig. 5a). TEM images of NPs where the molecules have been deprotected are shown in Fig. 5b, where NPs form chains. We have measured the inter-nanoparticle distances between two adjacent nanoparticles and obtain a variation of NP distance between 5.9 Å and 14 Å with an average distance of 10 Å. These measurements are in very good agreement with inter-NP distances obtained in the theoretical calculations.

### 1,6-Hexanedithiol in nanoparticle-nanoelectrode bridge platform

**Figure 6:**
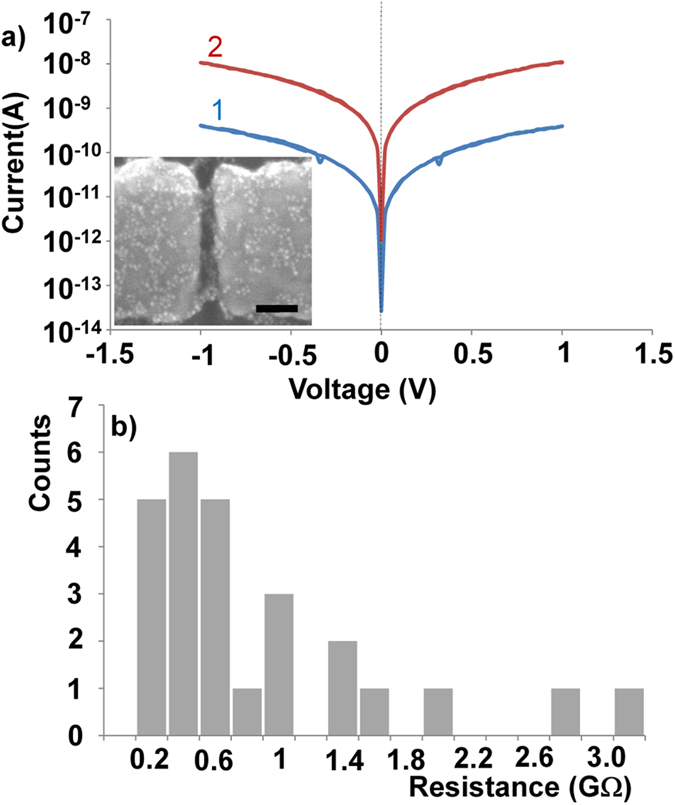
(**a**) Current-voltage (I–V) response of 1,6-hexandithiols coated gold nanoparticles in between nanoelectrode (inset: SEM of resultant device, scale bar:50 nm) after (1) trapping and (2) removal of trityl protective groups from ω-end of 1,6-hexanedithiol. (**b**) Linear scale resistance histogram (bin size = 200 MΩ) of 1,6-hexanedithiol in nanoparticle-nanoelectrode bridge platform after removal of trityl protecting group and establishing a chemisorbed metal-molecule junction.

To further verify the reproducibility and accuracy of our nanoparticle-nanoelectrode bridge platform, we have carried out similar studies with ω-trityl protected 1,6-hexanedithiol coated gold nanoparticles. In Fig. 6a, we have plotted the current-voltage (I–V) characteristics of junctions with these nanoparticles before and after removal of trityl protective groups. Similar as for the previous 1,8-octanedithiol case, clear increases in conduction are observed for the 1,6-hexanedithiol based devices. However, the devices before removal of protective groups demonstrate much higher currents than devices with ω-trityl protected 1,8-octanedithiol, which may be attributed to the smaller size of the 1,6-hexanedithiol molecules reducing the distance between two adjacent gold surfaces.

The measured resistance of devices after trapping of ω-trityl protected 1,6-hexanedithiol coated nanoparticles in between the nanoelectrodes (SEM inset Fig. 6a) is 5 GΩ as shown in Fig. 6a (1). The same device after deprotection demonstrates a 25 times higher current level as shown in Fig. 6a (2). The I–V curves are non-linear and the device demonstrates resistance of 179 MΩ. The resistance histogram of 26 devices having 1,6-hexanedithiol molecules in the device after deprotection is shown in Fig. 6b. 60% of such devices show device resistance values in range of 100 MΩ to 600 MΩ. Geometric mean resistance of all devices is found to 510 MΩ. Similarly to the devices based on 1,8-octanedithiol, four to five molecules of 1,6-hexanedithiol are required in series to bridge the gap between the nanoelectrodes. Thus, in a simple model, the average resistance of a single molecule attached to two gold surfaces in the device is estimated to about 100 MΩ, which is comparable to the value in literature^46^. It is shown here that these devices are highly reproducible and demonstrate stable current-voltage characteristics.

## Conclusion

We have demonstrated that the use of protection groups in the nanoparticle-molecule-nanoelectrode bridge platform followed by a deprotection step leads to a strong improvement of the reproducibility of such molecular electronics devices. The enhancement in reproducibility can be traced back to the formation of chemisorbed junctions linking two adjacent Au surfaces in the device. Thus, replacing the physisorbed metal-molecule junction with a chemisorbed metal-molecule junction leads to a decreased spread in the measured resistance histogram. The 2–3 orders of magnitude increase in conductivity after removal of protection group is in very good agreement with the results from DFT calculations. Also, the reduced spread of the resistance histogram by the order of magnitude is in good agreement with DFT calculations, still after chemisorption, conformational changes in the molecule and the metal-molecule interface can alter the conductivity by two to three orders of magnitude thus limiting the yield to the 55%. Thus we have shown that the nanoparticle-molecule-nanoelectrode bridge platform can be used for fundamental physical measurements on electrically interconnected molecules in AuNPs network. Moreover, since the platform can be easily transported and is built by conventional micro-and nano-structuring tools, it also can be upscaled to build applications based on current transport at a level of few molecule levels.

## Experimental Section

### Preparation of nanoparticles coated with trityle protected alkanedithiols solution

A solution of 1.90 mmol HAuCl_4 _× 3H_2_O is mixed with 1.90 mmol of the corresponding α,ω-bis(triphenylmethylthio)alkane in 20 ml of THF. The solution is vigorously stirred at 25 °C until the solution starts to become cloudy. To the resulting auburn solution, dropwise triethylsilane (1.90 mmol) is added at 25 °C that forms a purple solution immediately. 18 hours of stirring at 25 °C is done and the solution is filtered. Ethanol (500 ml) is added to precipitate gold nanoparticles. To remove unbound molecules, the resultant solution is filtered again and the precipitate is washed exhaustively with ethanol (3 × 300 ml). Benzene is added to solve the precipitate from the filter paper. After evaporation of the solvent the alkanedithiol-derivatised gold nanoparticles are obtained as a red solid. For trapping experiments, gold nanoparticles are dispersed in toluene.

### Preparation of Gold Nanoelectrodes

The combined techniques of electron-beam lithography and photography and ion beam milling are employed to prepare nanoelectrode platforms with gap sizes in the range of 20 nm by using the top-down approaches on 300 nm thick SiO_2_ thermally grown on silicon wafer^21^,^29^. Gold wire are patterned using a FEI XL30 Environmental Scanning Electron Microscope (ESEM) operated at an acceleration voltage of 30 kV is used to pattern gold wire with dimensions of 100 nm wide and 2 μm long. Gold deposition is carried out by resistive evaporation, where 2–3 nm thin layer of chromium is used as adhesion layer between gold and SiO_2_. To fabricate nanogaps, the highly focused beam (1pA) of Ga^+^ ions at an acceleration voltage of 30 kV is used to cut nanogaps 15–20 nm wide in the gold wire by usage of a FEI Strata DB235 FIB/SEM. 100 μm × 200 μm gold contact pads are prepared by the standard photolithography technique.

### Preparation of molecular electronic devices

Molecular electronic devices are prepared by dielectrophoretic trapping of ω-trityl protected alkanedithiol gold nanoparticles in between nanoelectrodes. It starts with the placement of a 10 μL drop of solution containing gold nanoparticles on top of the nanoelectrodes. The alternating voltage (AC) between 1.25 to 1.5 V peak-peak at frequency 1 MHz is applied for 45 to 60 second by using a Tektronix AFG 3102 Function generator. The device is later washed with de-ionized water to remove excessive solution.

### Electrical characterization of molecular electronic devices

Karl Suss probe station is used to make electrical connections to the contact pads. Current-voltage (I–V) measurements are recorded in ambient conditions in the same probe station using an Agilent B1500A semiconductor parameter analyzer. The whole measurement is done in a Faraday cage and in dark. The measurement setup is placed on top of a stone table to reduce mechanical vibrations. Current-voltage (I–V) measurements are recorded by making positive and negative voltage sweeps while measuring the corresponding currents.

### Structural characterization by electron microscopy

To observe approximate number of trapped nanoparticles in between nanoelectrodes, scanning electron microscopy is done by using Zeiss LEO 1550 after electrical characterization of all devices. For SEM imaging, the microscope is operated at 15 kV and inlens detector is utilized to record images.

In this study, transmission electron microscopy has been carried out in order to measure inter-nanoparticle distances and agglomeration before and after removal of the trityl protective groups from alkanedithiol in nanoparticle dispersion. Transmission electron microscopy was carried out using FEI Tecnai F30 ST at 300 kV. A 10 μL drop of ω-trityl protected 1,8-octanedithiol molecules coated gold nanoparticles solution was poured on TEM carbon grid and immediately blown-dried with nitrogen to prevent nanoparticle agglomeration due to drying. To remove the protective trityl groups, deprotection solution was added to the gold nanoparticle dispersion, after few minutes again a 10 μL drop was added on the carbon TEM grid and blow-dried with nitrogen, again in order to prevent agglomeration due to drying.

### Theory/calculation techniques

The theoretical calculations were performed using previously reported methods^23^,^47^. Using the density functional theory (DFT) based code SIESTA , the ODT adsorption structures both with/without protection groups/backbiting molecules were first optimized between two Au(111) surfaces. Full self-consistent transport calculations were performed on the relaxed structures based on nonequilibrium Green’s function (NEGF) transport theory as implemented in TranSIESTA^48^. All calculations were performed using the local-density approximation (LDA) for the exchange correlation potential, core electrons were modeled with Troullier–Martins soft norm-conserving pseudopotentials^49^ and the valence electrons were expanded in a double-zeta with polarization orbitals (DZP) basis set of local orbitals for all atoms except Au were a single-zeta polarized (SZP) were used^50^. In contrast, a calculation section represents a practical development from a theoretical basis.

## Additional Information

**How to cite this article**: Jafri, S. H. M. *et al.* Nano-fabrication of molecular electronic junctions by targeted modification of metal-molecule bonds. *Sci. Rep.* **5**, 14431; doi: 10.1038/srep14431 (2015).

## Supplementary Material

Supplementary Information

## Figures and Tables

**Table 1:** Summary of results at zero bias voltage obtained from DFT calculations of different molecular configurations.

Configuration	Gold to gold surface distance (Å)	Zero-bias conductance (Go)
1,8-Octanedithiols	15.3	30.5 × 10^−5^
ω-Trityl protected 1,8-octanedithiols	18.1	1.9 × 10^−5^
ω-Trityl protected 1,8-octanedithiosl with backbiting 1,8-octanedithiols	25.0	1.3 × 10^−7^
ω-Trityl protected 1,8-octanedithiols head-to-head	33.7	1.9 × 10^−9^
