# Supplementary material for: Nano-fabrication of molecular electronic junctions by targeted modification of metal-molecule bonds
Source: Sci Rep. 2015 Sep 23;5:14431. doi: 10.1038/srep14431 (PMC5155674; doi:10.1038/srep14431)
Supplement: Supplementary Information [file srep14431-s1.pdf]

Supplementary Information:

Nano-fabrication of molecular electronic junctions by targeted modification of metal-molecule bonds

S. Hassan M. Jafri[1,2], Henrik Löfås[3], Tobias Blom[1], Andreas Wallner[4], Anton Grigoriev[3], Rajeev Ahuja[2,5], Henrik Ottosson[4] and Klaus Leifer[1]*

1 Applied Materials Science, Department of Engineering Sciences, Uppsala University, Box 534, Uppsala SE-751 21, Sweden

2 Department of Electrical Engineering, Mirpur University of Science and Technology, Mirpur Azad Jammu and Kashmir 10250, Pakistan

3 Department of Physics and Astronomy, Uppsala University, Box 516, Uppsala SE-75120, Sweden

4 Department of Chemistry - BMC, Uppsala University, Box 576, Uppsala SE-751 23, Sweden

5 Applied Material Physics, Department of Materials and Engineering, Royal Institute of Technology (KTH), Stockholm SE-10044, Sweden

*Corresponding author email: Klaus.leifer@angstrom.uu.se

Current-Voltage characteristics of high resistance devices with a SEM image

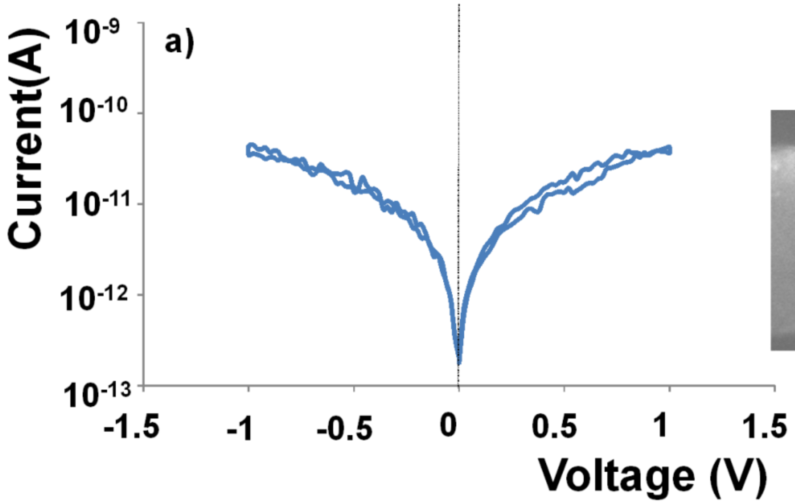

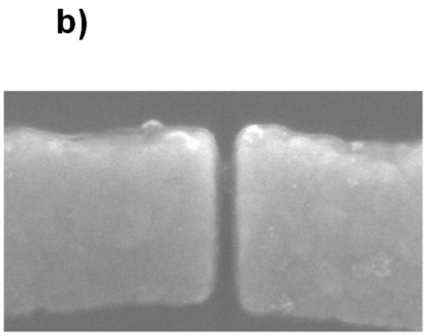

Figure S1: a) Current-voltage characteristics of high resistive device containing 1, 8-octanedithiol molecules in between nanoelectrode-nanoparticle bridge platform after removal of trityl protective groups b) respective SEM image Scale bar is 100nm

Current-Voltage characteristics of low resistance devices with a SEM image

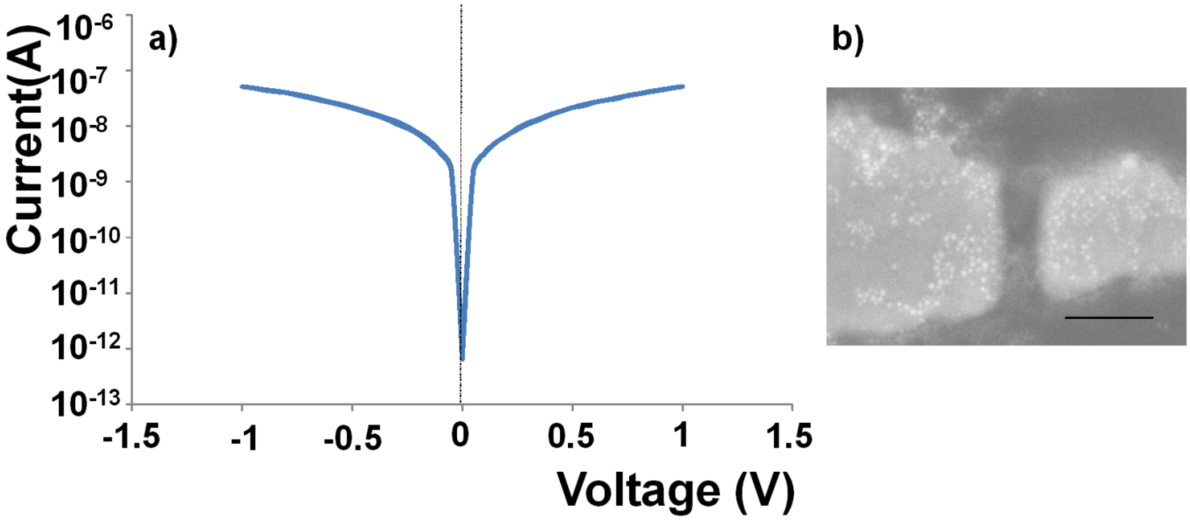

Figure S2: a) Current-voltage characteristics of high conductive device containing 1,8-octanedithiol molecules in between nanoelectrode-nanoparticle bridge platform after removal of trityl protective groups b) respective SEM image
